# Comparison of Different Risk Classification Systems in 558 Patients with Gastrointestinal Stromal Tumors after R0-Resection

**DOI:** 10.3389/fphar.2016.00504

**Published:** 2016-12-27

**Authors:** Michael Schmieder, Doris Henne-Bruns, Benjamin Mayer, Uwe Knippschild, Claudia Rolke, Matthias Schwab, Klaus Kramer

**Affiliations:** ^1^Department of Internal Medicine, Alb-Fils-KlinikenGöppingen, Germany; ^2^Department of General and Visceral Surgery, University Hospital UlmUlm, Germany; ^3^Institute of Epidemiology and Medical Biometry, University of UlmUlm, Germany; ^4^Dr. Margarete Fischer-Bosch Institute of Clinical PharmacologyStuttgart, Germany; ^5^Department of Pharmacy and Biochemistry, University of TübingenTübingen, Germany; ^6^Department of Clinical Pharmacology, University Hospital TübingenTübingen, Germany

**Keywords:** GIST, TKI, gastrointestinal stromal tumor, risk classification, prognosis, outcome

## Abstract

**Background:** Due to adjuvant treatment concepts for patients with R0-resected gastrointestinal stromal tumors (GIST), a reproducible and reliable risk classification system proved of utmost importance for optimal treatment of patients and prediction of prognosis. The aim of this study was to reevaluate the impact of five widely-applied and well-established GIST risk classification systems (i.e., scores by Fletcher, Miettinen, Huang, Joensuu, and TNM classification) on a series of 558 GIST patients with long-term follow-up after R0 resection.

**Methods:** Tumor size, mitotic count and site were used in variable combination to predict high- and low risk patients by the use of the five risk classification models. For survival analyses disease-specific survival, disease-free survival and overall-survival were investigated. Patients with initial metastatic disease or incompletely resectable tumors were excluded.

**Results:** All GIST classification models distinguished well between patients with high-risk and low-risk tumors and none of the five risk systems was superior to predict patient outcome. The models showed significant heterogeneity. There was no significant difference between the different risk-groups regarding overall-survival. Subdivision of GIST patients with very low- and low-risk appeared to be negligible.

**Conclusions:** Currently applied GIST risk classification systems are comparable to predict high- or low-risk patients with initial non-metastatic and completely resected GIST. However, the heterogeneity of the high-risk group and the absence of differences in overall survival indicate the need for more precise tumor- and patient-related criteria for better stratification of GIST and identification of patients who would benefit best from adjuvant tyrosine kinase inhibitor therapy.

## Introduction

Gastrointestinal stromal tumors (GIST) are the most common mesenchymal neoplasms of the gastrointestinal tract with an annual incidence of 7–20 per million (Tran et al., 2005; Nilsson et al., 2005; Tryggvason et al., 2007; Tzen et al., 2007; Steigen et al., 2008; Cassier et al., 2010), but their incidence might be underestimated (Choi et al., 2015). There is substantial evidence that GISTs differentiate similar to the gut pacemaker cells, the interstitial cells of Cajal (Barajas-López et al., 1989), suggesting an origin from these or their mesenchymal progenitor cells (Kitamura et al., 1998; Miettinen et al., 1999). While a majority of GISTs (~80%) are driven by gain-of-function mutations in the proto-oncogene *KIT* on chromosome 4q11-21 (Hirota et al., 1998; Kindblom et al., 1998; Sommer et al., 2003; Rubin et al., 2005), about 20% of GIST lack *KIT* mutations but either carrying gain-of-function mutations of the *KIT* homolog platelet-derived growth factor receptor alpha (*PDGFRA*), or wildtype for both genes. Most GISTs are diagnosed in the 6–7th decade of life (Miettinen et al., 2005; Woodall et al., 2009).

Complete surgical resection of the tumor is still the gold standard of treatment of patients with resectable localized disease. However, the impact of powerful tyrosine kinase inhibitors (TKI), like imatinib mesylate, as targeted therapy in GIST is increasing as adjuvant (Dematteo et al., 2009; Joensuu et al., 2012a) or supportive (palliative) (Blanke et al., 2008a,b) therapy in patients with high risk and advanced/unresectable disease. Though proven highly effective as first-line therapy for metastatic disease, a wide spectrum of TKI-related side effects and high socio-economic treatment costs (Rubin et al., 2010) make it necessary to establish better criteria for the decision making process. In addition to the TKI use as durable first-line treatment for metastatic GIST, guidelines of the European Society For Medical Oncology (ESMO) (The ESMO/European Sarcoma Network Working Group, 2014) and the National Comprehensive Cancer Network (NCCN) recommend the adjuvant use of TKI for patients with “a significant risk of relapse,” particularly those with high risk tumors but also for tumors with intermediate risk up to 3 years (Joensuu et al., 2012a). However, a precise definition of a “significant risk of relapse” is challenging as almost one half of patients in the intermediate- and high-risk group will not develop metachronous disease progression. Furthermore, the current ESMO guidelines (The ESMO/European Sarcoma Network Working Group, 2014) mainly recommend the use of the criteria of Miettinen and Lasota (2006), but in common clinical practice risk assessment depends strongly on the center-specific expertise of the respective pathologists and/or oncologists. Accordingly, there is a clinical need for a more reliable risk classification system that is simple to apply and able to stratify more precisely high-risk-, low-risk, and very-low-risk patients for progression of disease.

In the past, more than eight GIST classification systems have been established. The classification scores by Fletcher et al. (2002) and Miettinen and Lasota (2006) are most widely clinically accepted. Nevertheless, independent reevaluation even of these classification systems in lager cohorts is limited.

In 2002, Fletcher et al. proposed the first risk classification system for GIST which is currently termed as “NIH classification” by some authors (Fletcher et al., 2002). Based on size (the single largest dimension) and mitotic count in 50 high power fields (HPF), a four grade scale to predict biological behavior was proposed according to the former work of Franquemont et al. (Franquemont and Frierson, 1992). Subsequently, GISTs were divided into four groups with high, intermediate, low and very low risk of progression, thereby excluding a benign category. The usefulness of this classification has been proved in several studies (Nilsson et al., 2005; Andersson et al., 2006; Mucciarini et al., 2007; Rutkowski et al., 2007; Takahashi et al., 2007; Goh et al., 2008; Hassan et al., 2008; Joensuu et al., 2012b). Nevertheless, the area of 50 HPFs is imprecisely defined.

In 2006, Miettinen et al. published a new classification based on the evaluation of 1765 GIST of the stomach and 906 GIST of the small intestine (Miettinen et al., 2005, 2006; Miettinen and Lasota, 2006) which is also termed as the Armed Forces Institute of Pathology (AFIP) classification. The main criteria were also mitotic count and primary tumor size. However, based on the observation that gastric GISTs showed a much lower rate of aggressive behavior than comparable intestinal GISTs, Miettinen et al. introduced the anatomic site of the primary tumor as an additional criterion in risk assessment. They were also the first to specify the total area for mitotic counting (5 mm^2^). Using these three parameters, eight subgroups (group 1–6b) corresponding to five risk groups were established. In contrast to Fletcher et al. the Miettinen et al. classification considers a no risk/benign group. The main differences to the NIH-classification are a general downgrading of gastric tumors and an upgrading of a subset of non-gastric tumors. Therefore, in this classification, localization as a risk factor is more articulately appreciated.

In 2007, Huang et al. reevaluated NIH consensus criteria according to Fletcher et al. (2002), in *n* = 289 cases (Huang et al., 2007). They found no significant differences between the very low and low risk group, thereby merging both as “Level I” risk group. Due to a prognostic heterogeneity in the high-risk category of the NIH scheme, only GIST with a size >5 cm and >10 mitoses per 50 HPFs were rated as Level IV. The total area for mitotic counting was defined as 11.85 mm^2^.

Based on these new findings, Goh et al. proposed a revision of the AFIP-criteria (Goh et al., 2008) in 2008. They also merged very-low and low-risk patients to one group and introduced a very-high risk group, which corresponds to the high-risk group defined by Huang et al. (2007).

In 2008, Joensuu et al. published a large review on prognostic factors in GIST (Joensuu, 2008). Based on data by Takahashi et al. (2007) and Rutkowski et al. (2007), who found a negative prognostic effect of tumor rupture during surgery, he proposed a new risk classification and defined tumor rupture as an important prognostic parameter for high risk. The “modified NIH classification” was based on the classification presented by Fletcher et al. and Miettinen et al. The major differences to the original NIH system were the definition of tumors with exactly 5 cm diameter or 5 mitoses/50 HPFs, the consideration of tumor rupture as well as tumor site. However, the revised NIH classification by Joensuu neglected again the area of HPF. Later, Joensuu et al. published a comparative analysis of a pooled population-based cohort including 2560 patients from several trials (Nilsson et al., 2005; Mucciarini et al., 2007; Rutkowski et al., 2007; Steigen et al., 2007; Takahashi et al., 2007; Tryggvason et al., 2007; Braconi et al., 2008; Mazzola et al., 2008; Brabec et al., 2009) with a median follow-up time for patients alive of 4.0 years (Joensuu et al., 2012b). They investigated the predictive value of the NIH consensus criteria (Fletcher et al., 2002), the modified consensus criteria according to Joensuu (2008) and the AFIP criteria according to Miettinen and Lasota (2006). The authors concluded that the previously presented criteria identified high-risk patients at best which has been confirmed by other groups (Jang et al., 2014; Yanagimoto et al., 2015).

In 2010 the first TNM classification for GIST was published (Sobin et al., 2010). This system actually adopted the classification of Miettinen et al., including the definition of mitotic area which was defined as 5 mm^2^. However, the TNM classification has mainly focused on renaming the eight subgroups defined by Miettinen et al. to represent various tumor stages. A minor modification considered metastasis as a stage IV disease similar to other cancer types. The high-risk group introduced by Miettinen and Lasota (2006) corresponds to stage III. The ESMO guidelines do not recommend the use of this classification in its current form (The ESMO/European Sarcoma Network Working Group, 2014). Recently, Agaimy proposed an “integrated risk system” (Agaimy, 2013) by integration of the criteria of Miettinen et al., Joensuu as well as a “clinically malignant” category.

Finally, several authors have presented nomograms and heat maps for predicting the outcome where mostly tumor size, mitotic index and localization of the primary tumor are used either as continuous or as discrete variables (Gold et al., 2009; Rossi et al., 2011; Bischof et al., 2014).

Table 1 gives an overview of the different classification systems.

**Table 1:** **Overview of different risk classification systems for GIST considering different sites, tumor size and mitotic rates using uniform nomenclature**.

		**Gaster**						**Small intestine**					
**Size**	**Mitotic rate**	**Fletcher**	**Miettinen**	**Huang**	**Joensuu**	**TNM T**	**TNM S**	**Fletcher**	**Miettinen**	**Huang**	**Joensuu**	**TNM T**	**TNM S**
≤ 2 cm	≤ 5	I	0	I	I	T1	IA	I	0	I	I	T1	I
	>5 and ≤ 10	III	0	II	III	T1	II	III	IV	II	III	T1	IIIA
	>10	IV	0	III	IV	T1	II	IV	IV	III	IV	T1	IIIA
>2 and ≤ 5 cm	≤ 5	II	I	I	II	T2	IA	II	II	I	II	T2	I
	>5 and ≤ 10	III	III	II	III	T2	II	III	IV	II	III	T2	IIIB
	>10	IV	III	III	III	T2	II	IV	IV	III	IV	T2	IIIB
>5 and ≤ 10 cm	≤ 5	III	II	II	III	T3	IB	III	III	II	IV	T3	II
	>5 and ≤ 10	IV	IV	III	IV	T3	IIIA	IV	IV	III	IV	T3	IIIB
	>10	IV	IV	IV	IV	T3	IIIA	IV	IV	IV	IV	T3	IIIB
>10 cm	≤ 5	IV	III	IV	IV	T4	II	IV	IV	IV	IV	T4	IIIA
	>5 and ≤ 10	IV	IV	IV	IV	T4	IIIB	IV	IV	IV	IV	T4	IIIB
	>10	IV	IV	IV	IV	T4	IIIB	IV	IV	IV	IV	T4	IIIB
		**Duodenum**						**Colorectal**					
≤ 2 cm	≤ 5	I	0	I	I	T1	I	I	0	I	I	T1	I
	>5 and ≤ 10	III	IV	II	III	T1	IIIA	III	IV	II	III	T1	IIIA
	>10	IV	I	III	IV	T1	IIIA	IV	IV	III	IV	T1	IIIA
>2 and ≤ 5 cm	≤ 5	II	II	I	II	T2	I	II	II	I	II	T2	I
	>5 and ≤ 10	III	IV	II	III	T2	IIIB	III	IV	II	III	T2	IIIB
	>10	IV	IV	III	IV	T2	IIIB	IV	IV	III	IV	T2	IIIB
>5 and ≤ 10 cm	≤ 5	III	IV	II	IV	T3	II	III	IV	II	IV	T3	II
	>5 and ≤ 10	IV	IV	III	IV	T3	IIIB	IV	IV	III	IV	T3	IIIB
	>10	IV	IV	IV	IV	T3	IIIB	IV	IV	IV	IV	T3	IIIB
>10 cm	≤ 5	IV	IV	IV	IV	T4	IIIA	IV	IV	IV	IV	T4	IIIA
	>5 and ≤ 10	IV	IV	IV	IV	T4	IIIB	IV	IV	IV	IV	T4	IIIB
	>10	IV	IV	IV	IV	T4	IIIB	IV	IV	IV	IV	T4	IIIB
		**EGIST**						**Esophagus**					
≤ 2 cm	≤ 5	I	0	I	I	T1	I	I	0	I	I	T1	I
	>5 and ≤ 10	III	IV	II	III	T1	IIIA	III	IV	II	III	T1	IIIA
	>10	IV	IV	III	IV	T1	IIIA	IV	IV	III	IV	T1	IIIA
>2 and ≤ 5 cm	≤ 5	II	II	I	II	T2	I	II	II	I	II	T2	I
	>5 and ≤ 10	III	IV	II	III	T2	IIIB	III	IV	II	III	T2	IIIB
	>10	IV	IV	III	IV	T2	IIIB	IV	IV	III	IV	T2	IIIB
>5 and ≤ 10 cm	≤ 5	III	III	II	IV	T3	II	III	III	II	IV	T3	II
	>5 and ≤ 10	IV	IV	III	IV	T3	IIIB	IV	IV	III	IV	T3	IIIB
	>10	IV	IV	IV	IV	T3	IIIB	IV	IV	IV	IV	T3	IIIB
>10 cm	≤ 5	IV	IV	IV	IV	T4	IIIA	IV	IV	IV	IV	T4	IIIA
	>5 and ≤ 10	IV	IV	IV	IV	T4	IIIB	IV	IV	IV	IV	T4	IIIB
	>10	IV	IV	IV	IV	T4	IIIB	IV	IV	IV	IV	T4	IIIB

*0, None; I, Very Low; II, Low; III, Intermediate; IV, High*.

Meanwhile, several authors, as well as the current ESMO guidelines, have recommended the standardization of mitotic counting for GIST. The ESMO suggests a total area of 5 mm^2^ for counting mitotic figures based on retrospective analyses using AFIP microscopes, an approach which was not validated by independent studies. Depending on the field-of-view number (FOV) and the applied eyepiece, the field area per HPF (e.g., ~0.26 mm^2^ with FOV 23, ~0.33 mm^2^ with FOV 26)—and therefore the necessary number of HPF to count—differs from microscope to microscope. Ignoring this, mitotic rate might be consequently overestimated up to 3.1-fold (e.g., counting 50 instead of 16 HPFs) resulting in upgrading of patients' risk—at least in a number of cases.

With respect of the emerging initiative of standardization of established risk classification systems for GIST and standardized mitotic counting (Agaimy, 2010; Patel, 2011), the aim of this study was to re-evaluate the predictive value of relevant risk assessment tools for GIST in a series of 558 patients.

## Materials and methods

This study included patients with histologically confirmed diagnosis of GIST from the multi-center Ulmer GIST Registry—a multi-center network encompassing 18 oncological centers in South Germany—from 2006 to 2012. The patients were registered according to the User's Guide to Registries Evaluating Patient Outcomes and to the Strengthening (of) the Reporting of Observational Studies in Epidemiology (STROBE) Statement (Vandenbroucke et al., 2007a,b,c; Gliklich and Dreyer, 2010; Kramer, 2012). All cooperating centers are non-restricted, open hospitals implicating neither demographic nor social or clinical selection bias. Patients diagnosed before 2006 were registered retrospectively. Since 2006, registration and follow up were intended to be prospectively. After obtaining patients' consent, descriptive and clinical data were collected from medical records by a personal patient contact and/or contact with the treating physicians. The Ulmer GIST registry has been previously described in detail (Lott et al., 2015; Kramer et al., 2015a,b). Only patients with initial non-metastatic R0-resected gastric and small bowel GISTs were included in this analysis. Consequently, patients with primary metastatic disease and those with incomplete resectable tumors were excluded.

The following GIST risk classification models were applied in this study: Fletcher et al. (2002), Miettinen and Lasota (2006), Huang et al. (2007), Joensuu (2008), and the TNM-system (Sobin et al., 2010). Mitotic count areas have been considered so far as given: Miettinen and Lasota (2006) (5 mm^2^), Huang et al. (2007) (11.85 mm^2^), and the TNM classification (Sobin et al., 2010) (5 mm^2^). In accordance with the recommendation by Miettinen et al. to limit mitotic counting to 25 HPFs in wide-field-microscopes, a calculated area of 7.5 mm^2^ was used to re-evaluate the classification system of Fletcher et al. (2002) and Joensuu (2008) (revised NIH criteria).

Area-standardized mitotic rates were gained by calculation, only if a mitotic rate in 50 HPFs and at least 15 mm^2^ was provided. After calculation of the total counted area A using the field-of-view number f (diameter of the view field in millimeters measured at the intermediate image plane) and the magnification of the objective m_0_ = 40 in the formula $\text{A}=\left({\left(\frac{\text{f}}{2\times {\text{m}}_{0}}\right)}^{2}\times \pi \right)\text{m}{\text{m}}^{2}$, the absolute value of the mitotic rate was interpolated to 5 mm^2^ for the classifications according to Miettinen and Lasota (2006) and TNM (Sobin et al., 2010), to 7.5 mm^2^ for the classifications according to Fletcher et al. (2002) and Joensuu (2008), as well as to 11.85 mm^2^ for the classification according to Huang et al. (2007). Tumors were assigned to the respective risk group afterwards.

Throughout the manuscript we used an adapted uniform nomenclature. Most of the classification systems propose a four-point scale: high risk group, intermediate, low and very low risk group. With regard to the TNM classification, stage III was assigned to high-risk patients. For calculation of the predictive value of the highest risk category all other GIST risk groups (i.e., intermediate-, low-, and very low-risk group) were merged, collectively termed as “non-high risk group.”

The classification system by Woodall et al. (2009) could not be considered due to the lack of data on tumor grading. The nomograms by Gold et al. (2009), Rossi et al. (2011), and Bischof et al. (2014) resulting in continuous values were not considered in our study because all other classifications tools provide ordinal values. The proposed classification by Goh et al. (2008) was finally not considered, because in our setting (mainly comparing high and non-high) the results are quite similar to the classification according to Huang et al. (2007).

For survival analyses the following end-points were used: GIST-dependent death (disease-specific survival [DSS]), occurrence of recurrence or metastasis (disease-free survival [DFS]), as well as death in general (overall-survival [OS]). The high-risk group was compared to pooled data of the non-high-risk-groups. Moreover, survival analyses were performed between the non-high-risk-groups. To exclude an effect of TKIs (Imatinib or others), analyses were performed censoring all end-points and follow-up data after the beginning of TKI treatment (TKI-adjusted survival).

For the statistical analysis of DSS, DFS and OS, Kaplan-Meier-curves were compared using the log-rank test after Mantel-Cox. Additionally, Cox-regression analyses were applied reporting Hazard Ratios (HR) with corresponding 95% confidence intervals (CI). Data management was performed using a professionally developed graphical user interface based on a XML database. All statistical data was calculated using SPSS V19.0 (IBM Corp., New York, USA). The level of significance was set to α = 0.05.

The study was approved by the independent Ethics Committee of the University of Ulm (Study-No: 90 & 91/2006). All patients gave written informed consent in accordance with the Declaration of Helsinki.

## Results

### General data

The Ulmer GIST Registry comprises a total of 1106 patients with GIST acquired by multicentric cooperation. Finally, *n* = 558 patients matched the study criteria.

The male to female ratio was nearly balanced (male *n* = 268, female *n* = 290). Mean age at diagnosis was 65.8 years (SD ± 12.5) whilst the median age was 67.6 years (range: 14.7–94.8 years). Approximately one quarter of all patients (26.1%) were younger than 60 years, and 11.3% younger than fifty years. Patients younger than 40 years represented 3.7% of the cohort. The predominant proportion of study patients showed a primary location of GIST in the stomach (69.7%, *n* = 389). 169 patients (30.3%) revealed a location in the small intestine. By inclusion criteria only patients with R0-resection have been considered for analysis. TKIs were used in 10.4% (*n* = 58) of all cases.

TKI-adjusted follow-up of more than 3 months was available for 462 patients (82.8%). Mean and median follow-up time was 4.8 years (SD ± 3.8 years) and 4.2 years (range: 0.1–22.6 years), respectively. Disease progression (recurrence or metastasis) was found in 8.2% (*n* = 46) of all patients. Intraoperative tumor rupture was not reported. Table 2 summarizes clinical data as well as the distribution of patients regarding the risk-classification tools.

**Table 2:** **Demographic and clinicopathological data of 558 GIST patients of the Ulmer GIST registry**.

	**Percentage/n**		**Sum Cases**
**EPIDEMIOLOGY**			
Gender (male/female)	48.0/52.0	268/290	558
Mean age at diagnosis (yr ± SD)	65.8 (12.5)		547
Median age at diagnosis (yr, range)	67.6 (14.7; 94.8)		547
Mean follow-up time (yr ± SD)^§^	5.2 (3.7)/4.8 (3.8)		529
Median follow-up time (yr, range)^§^	4.8 (0.1; 22.6)/4.2 (0.1; 22.6)		529
**PATHOLOGY**			
Localisation (gaster/small intestine)	69.7/30.3	389/169	558
Mean tumor size (cm ± SD)	4.8 (4.1)		558
Median tumor size (cm, range)	3.8 (0.3; 32.0)		558
Histotype (spindle/epithelioid&mixed)	90.6/9.4	423/44	467
IHC KIT/CD117 (pos/neg)	97.5/2.5	507/13	520
IHC CD34 (pos/neg)	87.9/12.1	350/48	398
IHC Actin (pos/neg)	33.8/66.2	103/202	305
IHC Desmin (pos/neg)	13.4/86.6	39/253	292
IHC S100 (pos/neg)	14.2/85.8	43/260	303
**RISK CLASSIFICATION (STANDARDIZED)**			
Fletcher et al.^*1^	10.9/22.4/41.2/25.4	61/125/230/142	558
Huang et al.^*2^	10.6/2.5/21.3/65.6	59/14/119/366	558
Miettinen et al.^*3^	6.1/11.3/27.8/54.8	34/63/155/306	558
Joensuu^*1^	17.4/15.9/41.2/25.4	97/89/230/142	558
TNM Stage^*4^	6.1/11.3/82.6	34/63/461	558

§ , TKI-adjusted; SD, standard deviation; yr, years; TKI, tyrosine kinase inhibitor; IHC, immunohistochemistry;

*1 , (high, intermediate, low, very low);

*2 , (level IV, level III, level II, level I);

*3 , (high, intermediate, low, very low/none);

*4 *(Stage III, Stage II, Stage I)*.

### General survival analysis

GIST-dependent death was reported in 28 cases of whom half of them received TKI-therapy. Non-GIST related deaths were more common (*n* = 89), of which 14.5% had to be censored due to TKI-intake. In general, 1-, 3-, and 5-years DSS and OS rates were 98.9, 97.9, and 96.8%, 94.2, 87.2, and 81.9%, respectively. Metastasis or tumor recurrence occurred in 46 cases of which 10 had to be censored. Finally, the 1-, 3-, and 5-years DFS rates were 97.3, 94.8 and 93.2%, respectively (Table 3)

**Table 3:** **TKI-adjusted survival rates and Kaplan-Meier log-rank-tests for GIST patients for 1, 3, 5, and 10 years considering different risk classification systems and standardized mitotic counting**.

**Classification**		**DSS**	**OAS**	**DFS**
Fletcher et al.	High	92.5/89.6/89.6/83.2	90.5/84.5/77.2/57.3	85.4/71.4/65.3/60.7
	Intermediate	100.0/97.7/94.9/94.9	100.0/96.6/88.1/75.1	98.0/94.7/93.5/84.4
	Very Low/Low	99.4/99.0/99.0/98.5	92.8/84.6/80.5/70.8	98.7/97.9/96.9/96.0
	NonHigh	99.5/98.7/97.5/97.5	94.5/87.4/82.3/71.6	98.5/97.1/96.1/92.7
Miettinen et al.	High	86.3/80.9/80.9/60.7	86.9/75.9/69.0/25.9	77.7/61.7/56.1/56.1
	Intermediate	100.0/95.0/89.2/89.2	97.9/93.0/87.3/75.2	95.6/88.3/82.9/79.7
	Very Low/Low	99.5/99.2/98.8/98.8	94.1/87.1/81.9/71.5	98.7/97.5/96.7/92.9
	NonHigh	99.5/98.7/97.6/97.6	94.6/87.7/82.5/71.8	98.4/96.5/95.2/91.5
Huang et al.	Level IV	92.5/89.6/89.6/83.2	90.5/84.5/77.2/57.3	85.4/71.4/65.3/60.6
	Level III	100.0/100.0/97.5/n.a.	100.0/100.0/87.5/n.a.	90.0/80.0/70.0/n.a.
	Level I/II	99.5/98.6/ 97.8/97.8	94.4/87.1/82.2/ 71.3	98.8/97.6/96.8/93.3
	Level I-III	99.5/98.7/97.5/97.5	94.5/87.4/82.3/71.6	98.5/97.1/96.1/92.7
Joensuu	High	95.6/90.6/86.6/83.7	94.3/87.7/80.0/64.2	89.9/80.0/74.6/72.1
	Intermediate	100.0/100.0/100.0/100.0	100.0/98.4/89.7/76.5	98.6/95.6/95.6/82.8
	Very Low/Low	99.4/99.0/98.5/98.5	92.8/84.6/80.5/70.8	98.7/97.9/96.9/96.0
	NonHigh	99.5/99.2/98.8/98.8	94.1/87.1/82.2/71.8	98.7/97.5/96.7/92.9
TNM	Stage III	86.3/80.9/80.9/60.7	86.9/75.9/69.0/25.9	77.7/61.7/56.1/56.1
	Stage II	100.0/95.0/89.2/89.2	97.9/93.0/87.3/75.2	95.6/88.3/82.9/79.7
	Stage I	99.5/99.2/98.8/98.8	94.1/87.1/81.9/71.5	98.7/97.5/96.7/92.9
	Stage I/II	99.5/98.7/97.6/97.6	94.6/87.7/82.5/71.8	98.4/96.5/95.2/91.5
Overall		98.9/97.9/96.8/96.2	94.2/87.2/81.9/70.4	97.3/94.8/93.2/89.7
Classification		p (log-rank-test)	p (log-rank-test)	p (log-rank-test)
Fletcher et al. (High vs. Non-High)		***p* < 0.001**	*p* = 0.480	***p* < 0.001**
Miettinen et al. (High vs. Non-High)		***p* < 0.001**	***p* = 0.025**	***p* < 0.001**
Huang et al. (Level IV vs. I-III)		***p* < 0.001**	*p* = 0.475	***p* < 0.001**
Joensuu (High vs. Non-High)		***p* < 0.001**	*p* = 0.850	***p* < 0.001**
TNM (Stage III vs. Stage I/II)		***p* < 0.001**	***p* = 0.025**	***p* < 0.001**

*DSS, disease-specific survival; OAS, overall survival; DFS, disease-free survival*.

*Results with significant p-values are marked as bold*.

### Survival analysis of different classifications

**Figure 1:**
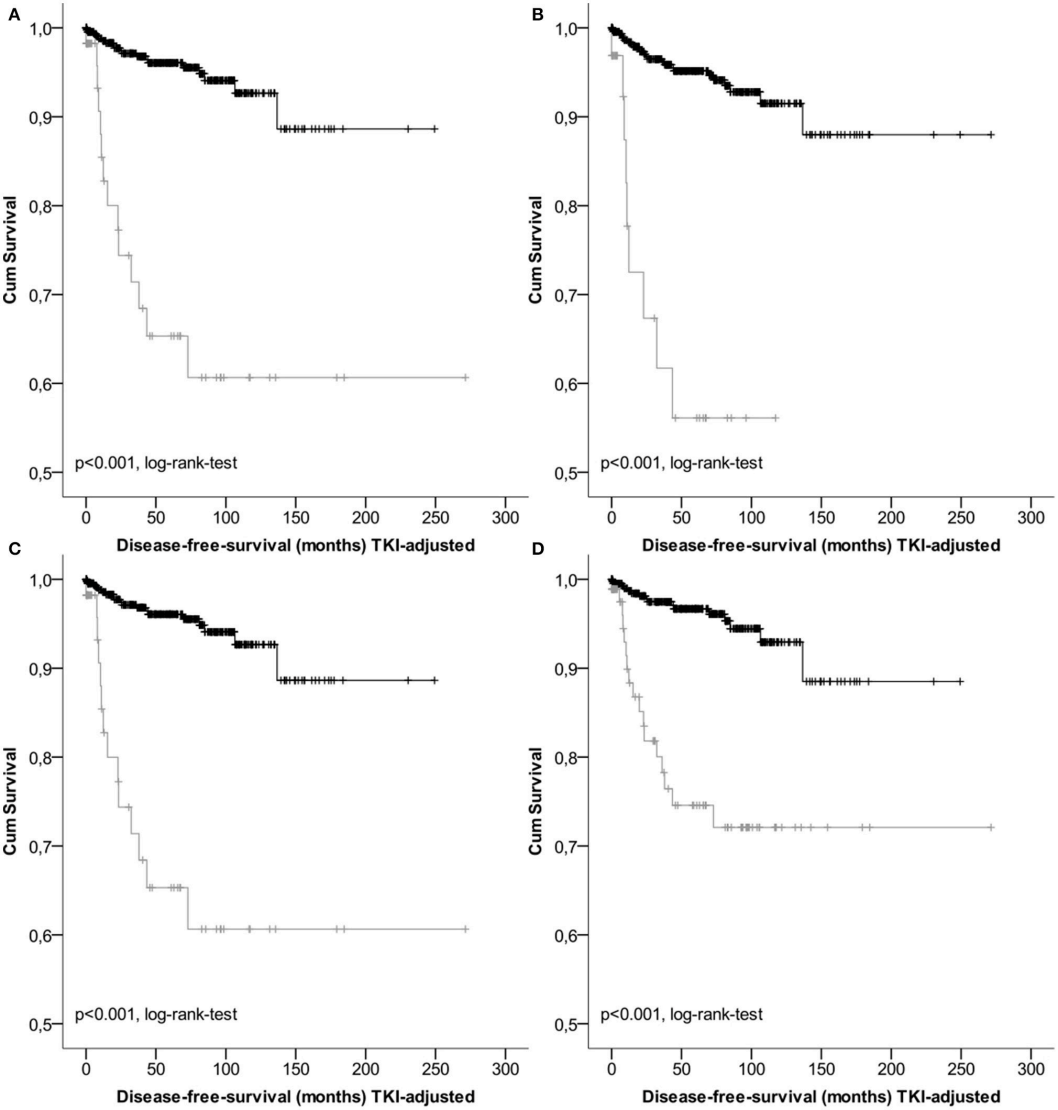
**Kaplan-Meier Plots of disease-free-survival of the high risk group (gray) and the non-high risk group (black) regarding different classification systems (A**, GIST classification acc.to Fletcher et al.; **B**, acc. to Miettinen et al./TNM; **C**, acc. to Huang et al.; **D**, acc. to Joensuu).

**Figure 2:**
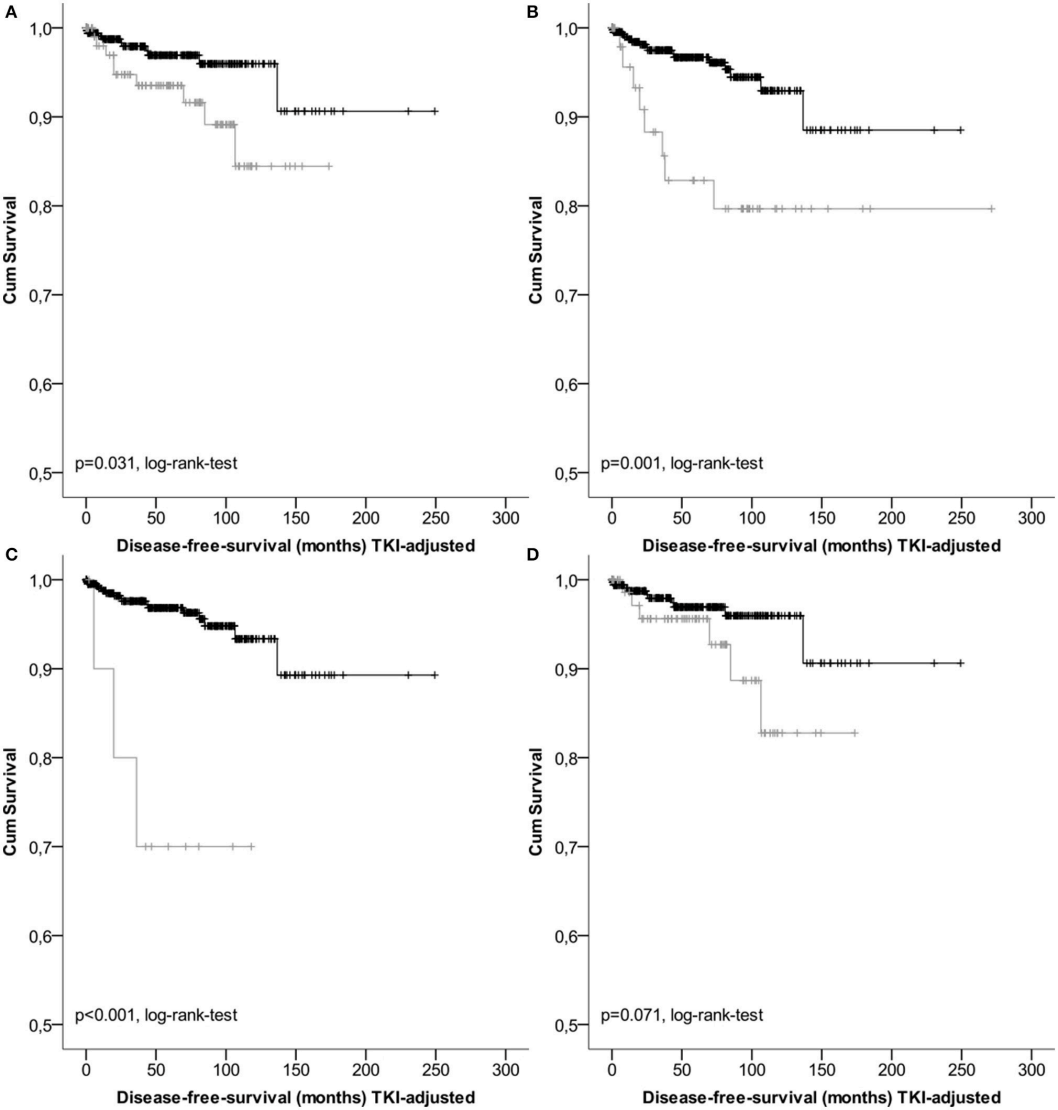
**Kaplan-Meier Plots of disease-free-survival for patients of the intermediate risk group (gray) and the low/very low risk group (black) regarding different classification systems (A**, GIST classification acc.to Fletcher et al.; **B**, acc. to Miettinen et al./TNM; **C**, acc. to Huang et al.; **D**, acc. to Joensuu).

Results of survival analysis comparing the following groups (high- vs. non-high risk, intermediate vs. high risk as well as vs. low/very low) are given in Table 3, indicating significant inter-group differences (*p* < 0.001 regarding DSS as well as DFS, log-rank-tests) except for the classification according to Joensuu. Lowest survival rates for the high-risk group were obtained by the classifications according to Miettinen and Lasota (2006) and the corresponding TNM-classification (Sobin et al., 2010). 1-, 3-, and 5-years DSS and DFS rates were 86.3, 80.9, and 80.9%, as well as 77.7, 61.7, and 56.1%, respectively. Corresponding Kaplan-Meier-Plots are given in Figures 1, 2. Terminal events in non-high risk groups regarding recurrence of disease or metastasis were less common and appeared predominantly in patients allocated to the intermediate risk group. 5-year DFS rates of the intermediate risk groups ranged between 70.0 and 95.6%. By contrast, 5-years DFS rates in the combined very low/low-risk groups ranged between 96.7 and 96.9%.

**Figure 3:**
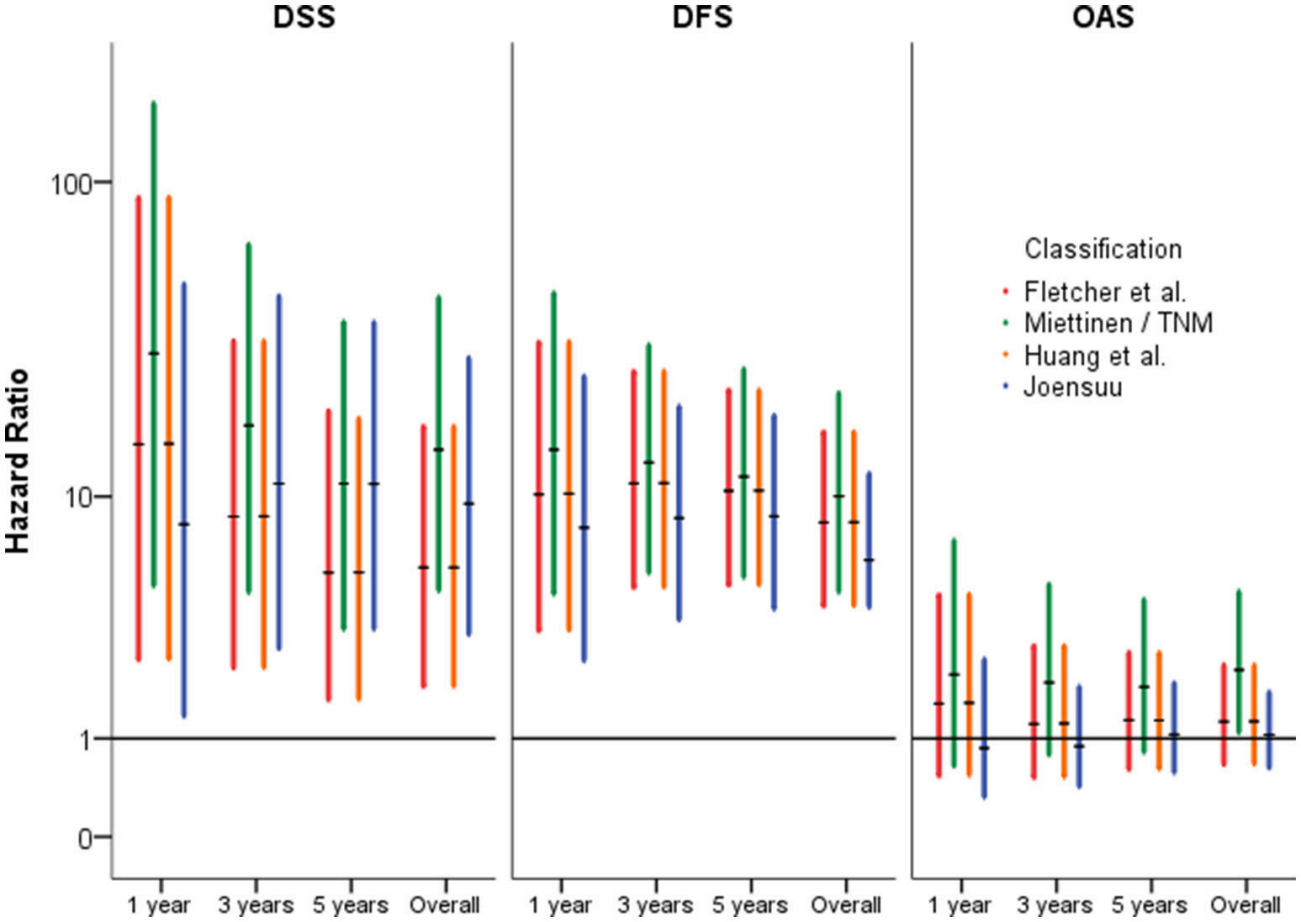
**Forest-Plots of Hazard-Ratios of different classification systems (high vs. non-high) regarding disease-specific- (DSS), disease-free- (DFS), and overall-survival (OAS) (y-axis logarithmic)**.

Not only the classification of patients with high-risk of progression is essential for novel treatment options, but also of patients with lower risk. Hazard Ratios may be more reliable to assess the clinical impact of classification systems than sensitivity and specificity. For prediction of tumor-dependent death, recurrence of disease and/or metastasis, the classification according to Miettinen and Lasota (2006) and consistently the TNM classification (Sobin et al., 2010) revealed highest reliability. For example, 5-year Hazard ratios for DSS and DFS were HR = 11.04 [3.317; 36.742] (*p* < 0.001) and HR = 11.662 [5.224; 26.031] (*p* < 0.001), respectively. However, due to overlapping confidence intervals, none of the considered classification models resulted in superior rating. These results are presented in Table 4 and are graphically given as Forest-Plots in Figure 3. Finally, regarding overall survival no significant differences were found between the highest and the lower risk groups except for the Miettinen and Lasota (2006) classification (equivalent to TNM system, Sobin et al., 2010; *p* = 0.025, log-rank-test, Table 3).

**Table 4:** **Hazard ratios (HR) for disease-specific survival (A), overall-survival (B) and disease-free survival (C) considering different risk classifications systems and standardized mitotic counting**.

		**HR 1y**	**HR 3y**	**HR 5y**	**HR 10y**	**Overall**
A	Fletcher et al. (High vs. Non-High)	14.89 [2.48; 89.21]	8.55 [2.29; 31.89]	5.43 [1.63; 18.06]	6.72 [2.19; 20.57]	5.66 [1.88; 16.99]
	Miettinen et al. (High vs. Non-High)	**29.16 [4.86; 174.74]**	**17.15 [4.59; 64.05]**	**11.04 [3.32; 36.74]**	**14.3 [4.66; 43.9]**	**14.3 [4.66; 43.9]**
	Huang et al. (Level IV vs. Level I-III)	14.96 [2.49; 89.59]	8.57 [2.29; 31.96]	5.44 [1.63; 18.09]	6.73 [2.2; 20.61]	5.67 [1.89; 17.02]
	Joensuu (High vs. Non-High)	8.04 [1.34; 48.14]	11.04 [2.76; 44.19]	11.03 [3.32; 36.65]	12.11 [3.72; 39.38]	9.45 [3.16; 28.27]
	TNM-classification (Stage III vs. Stage I/II)	**29.16 [4.86; 174.74]**	**17.15 [4.59; 64.05]**	**11.04 [3.32; 36.74]**	**14.3 [4.66; 43.9]**	**14.3 [4.66; 43.9]**
B	Fletcher et al. (High vs. Non-High)	1.55 [0.54; 4.49]	1.21 [0.52; 2.83]	1.27 [0.61; 2.65]	1.38 [0.73; 2.59]	1.25 [0.66; 2.34]
	Miettinen et al. (High vs. Non-High)	2.13 [0.64; 7.08]	1.96 [0.78; 4.92]	1.87 [0.81; 4.32]	2.24 [1.08; 4.63]	2.24 [1.08; 4.63]
	Huang et al. (Level IV vs. Level I-III)	1.57 [0.54; 4.52]	1.22 [0.52; 2.84]	1.27 [0.61; 2.66]	1.38 [0.73; 2.6]	1.25 [0.67; 2.35]
	Joensuu (High vs. Non-High)	0.86 [0.32; 2.5]	0.89 [0.42; 1.88]	1.05 [0.56; 1.96]	1.13 [0.67; 1.91]	1.05 [0.62; 1.77]
	TNM-classification (Stage III vs. Stage I/II)	2.13 [0.64; 7.08]	1.96 [0.76; 4.92]	1.87 [0.81; 4.32]	2.24 [1.08; 4.63]	2.24 [1.08; 4.63]
C	Fletcher et al. (High vs. Non-High)	10.16 [3.27; 31.58]	11.06 [4.79; 25.55]	10.45 [4.9; 22.26]	8.72 [4.33; 17.55]	8.16 [4.08; 16.3]
	Miettinen et al. (High vs. Non-High)	**14.31 [4.52; 45.25]**	**12.96 [5.42; 31.01]**	**11.66 [5.22; 26.03]**	**10.02 [4.61; 21.81]**	**10.02 [4.61; 21.81]**
	Huang et al. (Level IV vs. Level I-III)	10.22 [3.29; 31.74]	11.09 [4.8; 25.62]	10.47 [4.91; 22.31]	8.73 [4.34; 17.58]	8.17 [4.09; 16.33]
	Joensuu (High vs. Non-High)	7.83 [2.45; 24.7]	8.45 [3.61; 19.78]	8.57 [3.97; 18.48]	6.49 [3.23; 13.01]	6.02 [4.04; 11.94]
	TNM-classification (Stage III vs. Stage I/II)	**14.31 [4.52; 45.25]**	**12.96 [5.42; 31.01]**	**11.66 [5.22; 26.03]**	**10.02 [4.61; 21.81]**	**10.02 [4.61; 21.81]**

*Results with significant p-values are marked as bold*.

## Discussion

Since the discovery of activating kinase mutations as key targets for cancer therapy and the definition of GIST as a well-defined clinicopathological and molecular entity, so far more than eight risk classification systems have been established. However, most of them have not been re-evaluated in larger independent studies. While there is no doubt about the relevance of potent treatment strategies in case of metastatic GIST, there is currently minor consensus which treatment option may be best for the subgroup of non-metastatic and R0-resected GIST. Therefore, the aim of the present study was to elucidate comparatively the five most relevant risk GIST assessment models related to standardized clinical endpoints in our independent previously characterized GIST cohort.

With regard to the clinical and demographic parameters, our study cohort was not fully comparable to previously published large series (Miettinen et al., 2005, 2006; Woodall et al., 2009), because high-risk GIST were often metastasized at time of diagnosis, consecutively resulting in exclusion of these cases for our analysis. Thus, an underrepresentation of cases with metastasis during follow-up may be the consequence indicated by the high DFS rate of our cohort within the follow-up interval of 10 years (89.7%, Table 3). Five years DFS rates ranged from 74.6 to 56.1% for the high-risk group. These results are comparable to recent findings by Bischof et al., who reported 1−, 3−, and 5-year DFS rates of 95, 83, and 74% (Bischof et al., 2015). Furthermore, Kim et al. found a 5-year DFS rate of 74.9% in high-risk patients with gastric GIST (Kim et al., 2015). However, in their study, 2.8% had metastasis prior to operative procedures, 3.7% of all tumors were not R0-resected and at least 10.2% of all patients received a TKI, which might restrict comparability. A comparison to largest unbiased studies by Miettinen et al. (2005, 2006) is limited due to the lack of definite follow-up times and survival rates. However, 5-years DFS rates seem to be comparable to the reference study (Miettinen and Lasota, 2006) with given relapse rates for gastric GIST of 55–86% in high-risk patients (61.9% in our study) and 12–16% for the intermediate-risk group (20.2% our study). Considering GIST of the small intestine (except of the duodenum), Miettinen et al. found relapse rates of 52–90% (33.7% our study) for the high-risk group and about 24% in the intermediate-risk group (15.8% our study; Miettinen and Lasota, 2006).

Tumor-dependent death and recurrence of disease or metastasis were predicted best by the classification of Miettinen and Lasota (2006). However, as consequence of overlapping confidence intervals, no classification was superior to the others. (Table 4, Figures 1, 3).

Regarding lower risk-groups, survival rates for DSS and DFS with follow-up to 5 years ranged from 97.5 to 98.8% and from 95.2 to 96.7%, respectively (Table 3). As expected patients with intermediate risk showed a significant worse outcome compared to the DFS rates of above 95% for the combined groups with very low and low risk (Figure 2). Therefore, based on the data presented it appears to be feasible and justified to combine very low and low risk patients.

Finally, most interesting are the data regarding OS. Within a follow-up of 5 years, there was no significant difference between the high-risk- and the non-high-risk group, except for classification according to Miettinen et al. (Table 3). This indicates a prognostic relevance of GIST independent factors—at least for patients with initially non-metastatic GIST. In our study, non-GIST-related deaths were more common (*n* = 89), which corresponds to 76.1% of all lethal events. Thus, in the light of a mean age at diagnosis of 65.8 years (SD ± 12.5), secondary neoplasia (Giuliani and Bonetti, 2015; Murphy et al., 2015; Kramer et al., 2015b) or other age-related factors, like cardiovascular diseases may contribute substantially to OS. Therefore, further studies with representative follow-up times are required.

Limitation of the presented data might be that the area-standardized mitotic rates were gained by interpolation. Because mitotic counting is recommended to be initiated in areas with the highest proliferative activity, the used method could underestimate the mitotic rate at least in specimens with distinct inhomogeneous distribution of mitoses. However, only 34 cases (5.8%) of the whole cohort showed a mitotic rate ranging from 5 to 20 per 50 HPFs and a size below 10 cm, where calculation might have had an influence on the final risk allocation anyway.

## Synopsis of data and conclusion

Our data demonstrate that none of the five GIST risk systems analyzed in our study was superior to predict outcome of patients with initial non-metastatic and completely resected GIST. Thus, all these risk classification systems appear to be valid and feasible for clinical application. We confirmed in our study that indeed high-risk patients have had highest risk to develop recurrence of disease or metastasis resulting in a higher rate of tumor dependent death. Nevertheless, also patients of the intermediate-risk groups showed partly significant relapse rates within 5-year follow-up. Subdivision of GIST patients with very low- and low-risk appears to be negligible. Finally, it is noteworthy that a small subset of patients with low-risk GISTs developed recurrence of disease or metastasis within an interval of 10 years. The most interesting fact however, seems to be the absence of differences regarding OS between the different risk groups in the majority of the investigated classification systems.

In summary the analysis of classification systems supports the fact that tumors subsumed under the rubric GISTs represent a heterogeneous group of neoplasms that substantially differ in disease pathogenesis with consequences for tumor progression and clinical outcome. Heterogeneity in GISTs might not only be influenced by molecular, but also non-genetic factors such as age, gender, tumor site, and syndromic occurrence. An important question arises whether a GIST classification system in the future would be able to consider all important predictive factors? Of course, standardization of such a classification system with international consensus is obligate incorporating also a precise description of valid methods for mitotic counting (i.e., defining the best cut-off area and cut-off mitotic index based on validated prognostic studies). At present, current treatment strategies remain to be used, as long as there are no more obvious data on potential, additional risk factors regarding prognostic impact.

## Author contributions

The contributions of each author to the manuscript are: MiSc and KK conceived and designed the study. MiSc, KK, and CR were involved in the data acquisition. MiSc, BM, KK, MaSc, DH, and UK contributed to data analysis and interpretation. MiSc, KK, and MaSc contributed to the writing of the manuscript. All authors read and approved the final manuscript.

## Funding

MaSc was supported in part by the Robert Bosch Stiftung, Stuttgart, German and the IZEPHA grant Tübingen-Stuttgart. KK was supported by the SAGST grant p11496 (Darmstadt).

### Conflict of interest statement

The authors declare that the research was conducted in the absence of any commercial or financial relationships that could be construed as a potential conflict of interest.
